# Quackery in Georgia

**Published:** 1899-12

**Authors:** 


### *EDITORIAL NOTES AND COMMENTS.*

The Business office of THE JOURNAL-RECORD is 305, 306 Fitten Building.

The Editorial office is 318-319-320 The Prudential.

Address all Business communications to Dr. M. B. Hutchins, Mgr.

Make remittances payable to THE ATLANTA JOURNAL-RECORD OF MEDICINE.

On matters pertaining to the Editorial and Original Communications address Dr. Bernard Wolff, Atlanta.

Reprints of original articles will be furnished at cost price. Requests for the same should always be made *on the manuscript*.

We will present, post-paid, on request, to each contributor of an original article, twenty (20) marked copies of THE JOURNAL-RECORD containing such article.

### QUACKERY IN GEORGIA.

IT was hoped that after the passage of the bill through the State legislature, appointing a Medical Examining Board whose duty it was to see that only legally qualified physicians were allowed to practice, that Georgia would be one of the banner States as far as the standard of the medical profession was concerned. It was certainly a step in the right direction, but unfortunately the profession does not seem now to be in any better condition than it was before the passage of the law. We have previously called attention to this deplorable state of affairs, but we believe that it is of importance enough to continue bringing before the profession, and to see if something cannot be done to free the State from so much quackery and illegally qualified practitioners. The trouble is in our State Board of Medical Examiners. We dislike to say so, but nevertheless it is true. After a hard fight we succeeded in getting the legislature to pass this bill requiring all practitioners of medicine to stand examination before they would be allowed to practice in this State. The law is clearly defined. Twice a year the Board of Examiners hold a session to examine candidates who present themselves. This done they seem to think that their work is over, and yet right under their noses men will open up offices and practice medicine who, perhaps, have never even registered. Unless the law is made of more effect, the guilty culprits brought before a court of justice, then we might as well abolish all examinations for admission into practice in the State. If the State Board of Examiners will not have such punished, then the profession of the whole State should unite to employ legal counsel to prosecute these guilty ones. Ours is not the only State which suffers from the same practices. West Virginia is suffering in the same manner, but the profession in that State has taken the matter firmly in hand, to be dealt with accordingly. Here is what the *New York Medical Journal* says: "Although the State of West Virginia has a stringent law denying the right of practicing medicine to all persons who have not a license as a result of passing the State examination, there are many quacks there who seek to evade the law on the plea that they are not practitioners of medicine. Prominent among them are the 'osteopathists,' who profess to treat all diseases by manipulation, and claim immunity from the medical practice law on the ground that they do not write prescriptions, administer drugs, or perform surgical operations. Common sense gives the lie to their contention, and judicial decisions have done the same." A committee has been appointed by the medical society of this State to take up the matter of seeing that all are legally qualified.

"On September 7th, the committee authorized its chairman to send a circular letter to every physician in the State asking him to contribute a dollar to a fund for procuring counsel and meeting other expenses incident to enforcing the State medical laws . . . While they are not criticizing any 'system' of medicine, they are seeking to compel all practitioners of medicine to conform to the law." This action of the medical profession in the State of West

Virginia might well be imitated in this State, and we trust that the matter will be brought up at the next meeting of the State Medical Association in April. We are speaking largely, of course, locally, but just as quackery and illegally practicing physicians exist in Atlanta, so we imagine they exist in all the other larger cities of the State. In Atlanta the "Osteopath" has opened his office, and like every other "fad" in this city, is being overwhelmed with customers. The "Christian Scientists" pursue their healing art in defiance of all the laws of the State, charging for professional services just as the "family doctor," and yet what Christian Scientist has ever stood a medical examination and been given a license from the State? There has just been opened an Infirmary of Psychology and Healing by Hypnotism. We understand that their charges are very high, and therefore only the rich can afford such "ennui luxuries." Here is another case where the authorities might see if the proper license from the State Medical Examining Board has been obtained.

Every few days we see where some new medical company has opened offices where patients may receive treatment for every disease to which man is heir, and yet Georgia has a law just to protect the medical profession, and put all comers on the same footing. We are not criticizing any "system" of medicine, but we would compel all practitioners of medicine to conform to the law. As things exist now quacks and charlatans may come in and practice unmolested, because there is no one who will look into the matter, and compel such to conform to the law. Nor do we wish to make any stringent criticism upon the present Board of Examiners, but simply to urge the necessity of the board's taking cognizance of the presence of these illegally qualified practitioners and institutions and to have such prosecuted under the laws of the State. Some one must do this if the law is to be regarded with any respect, and we certainly think that it is the duty of the Examining Board to bring proper legal punishment to the offenders. If it is not its duty, then whose duty is it, and what is the necessity for such a law? The reputable physicians throughout the State must take this matter up again if we wish to rid Georgia of quacks, faith-healers, charlatans, etc.

